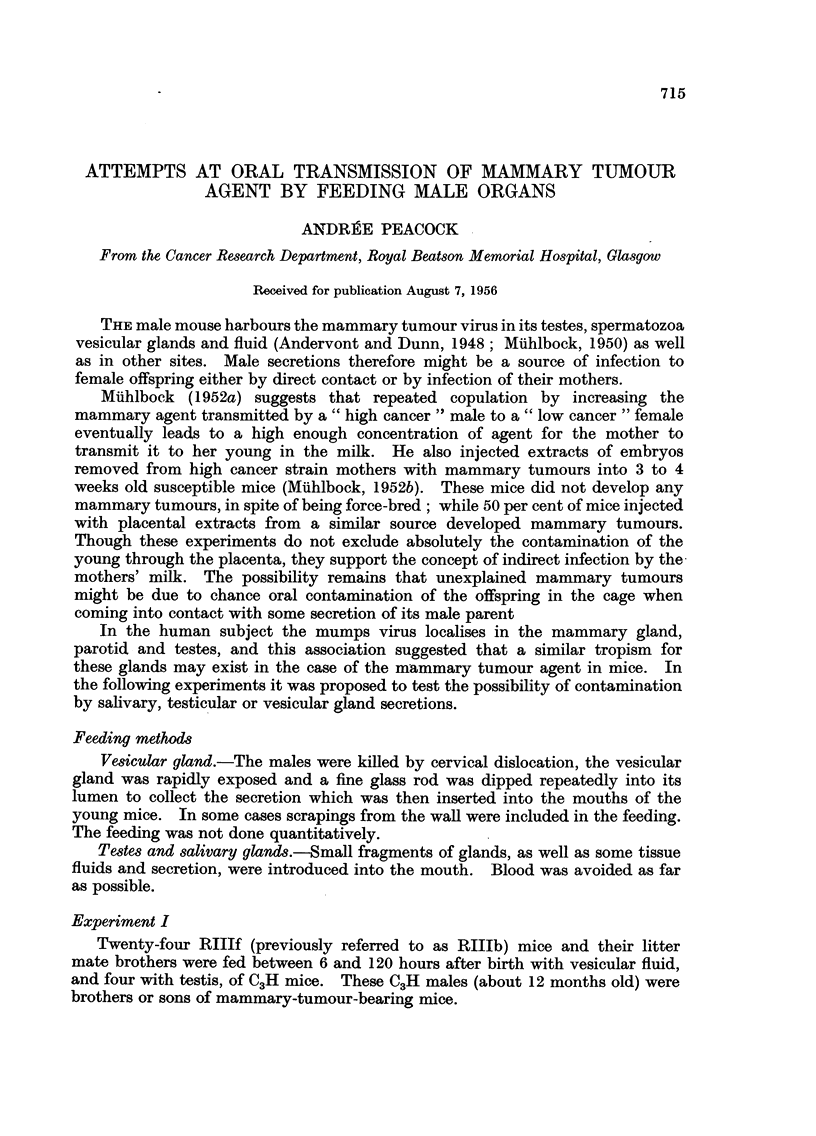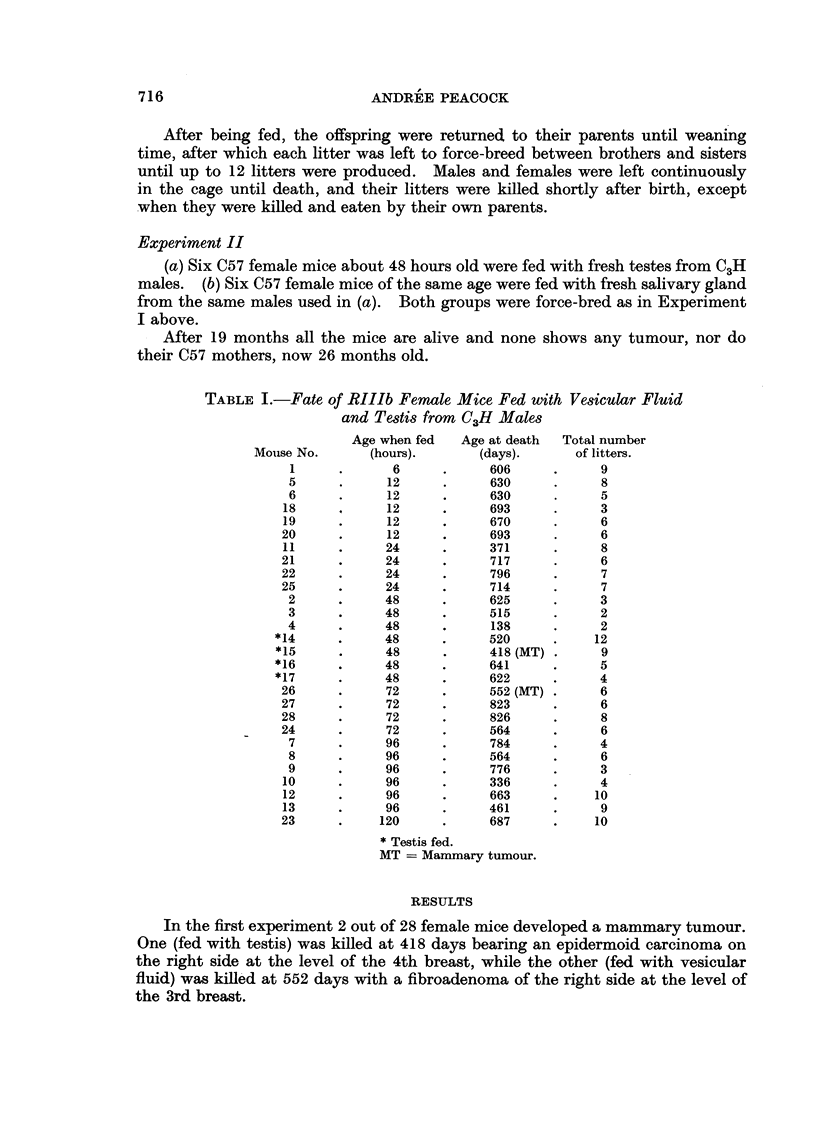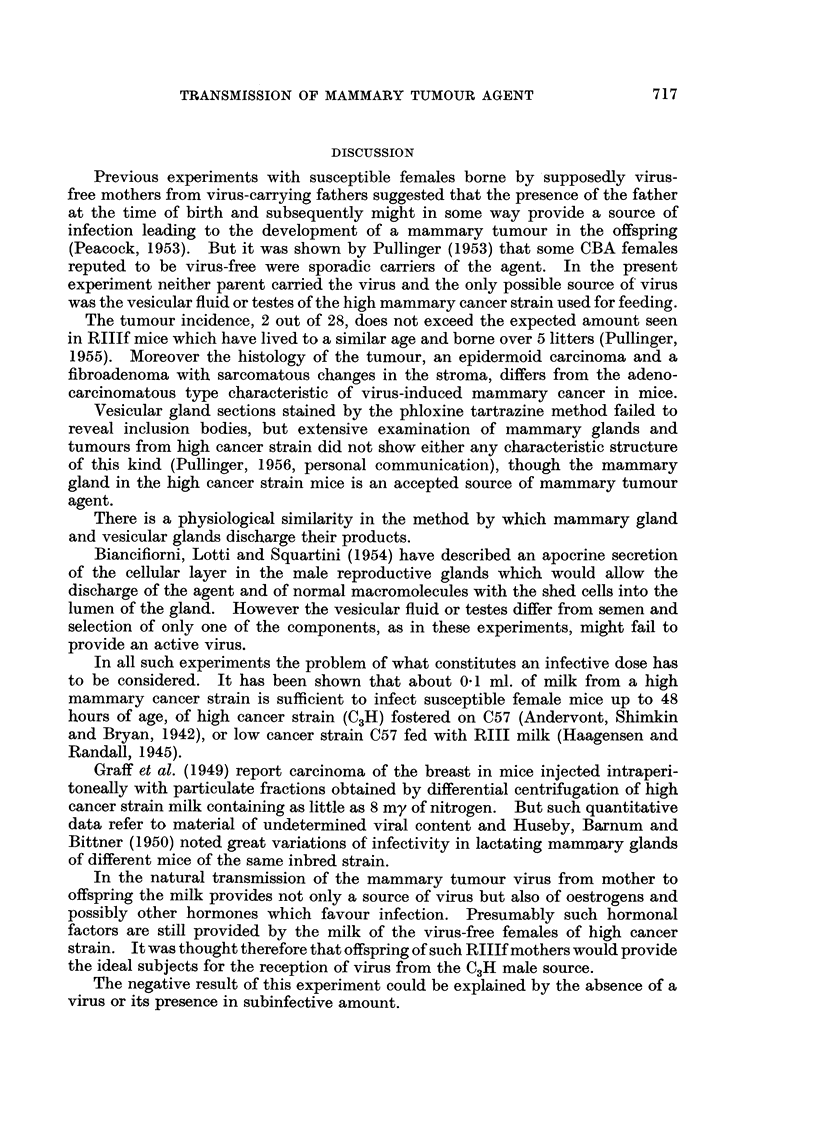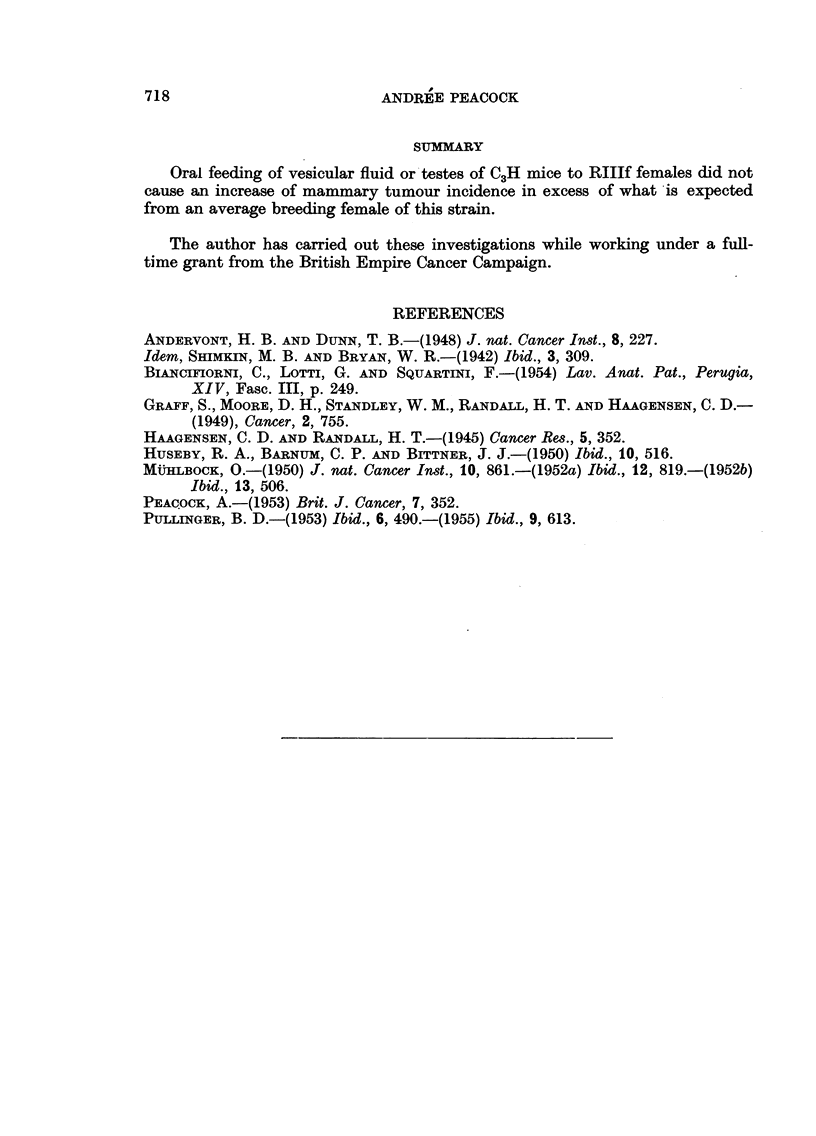# Attempts at Oral Transmission of Mammary Tumour Agent by Feeding Male Organs

**DOI:** 10.1038/bjc.1956.86

**Published:** 1956-12

**Authors:** Andrée Peacock


### ATTEMPTS AT ORAL TRANSMISSION OF MAMMARY TUMOUR AGENT BY FEEDING MALE ORGANS

ANDRÉE PEACOCK

*From the Cancer Research Department, Royal Beatson Memorial Hospital, Glasgow*

Received for publication August 7, 1956

THE male mouse harbours the mammary tumour virus in its testes, spermatozoa vesicular glands and fluid (Andervont and Dunn, 1948 ; Mühlbock, 1950) as well as in other sites. Male secretions therefore might be a source of infection to female offspring either by direct contact or by infection of their mothers.

Mühlbock (1952*a*) suggests that repeated copulation by increasing the mammary agent transmitted by a " high cancer " male to a " low cancer " female eventually leads to a high enough concentration of agent for the mother to transmit it to her young in the milk. He also injected extracts of embryos removed from high cancer strain mothers with mammary tumours into 3 to 4 weeks old susceptible mice (Mühlbock, 1952*b*). These mice did not develop any mammary tumours, in spite of being force-bred ; while 50 per cent of mice injected with placental extracts from a similar source developed mammary tumours. Though these experiments do not exclude absolutely the contamination of the young through the placenta, they support the concept of indirect infection by the mothers' milk. The possibility remains that unexplained mammary tumours might be due to chance oral contamination of the offspring in the cage when coming into contact with some secretion of its male parent

In the human subject the mumps virus localises in the mammary gland, parotid and testes, and this association suggested that a similar tropism for these glands may exist in the case of the mammary tumour agent in mice. In the following experiments it was proposed to test the possibility of contamination by salivary, testicular or vesicular gland secretions.

### *Feeding methods*

*Vesicular gland.*—The males were killed by cervical dislocation, the vesicular gland was rapidly exposed and a fine glass rod was dipped repeatedly into its lumen to collect the secretion which was then inserted into the mouths of the young mice. In some cases scrapings from the wall were included in the feeding. The feeding was not done quantitatively.

*Testes and salivary glands.*—Small fragments of glands, as well as some tissue fluids and secretion, were introduced into the mouth. Blood was avoided as far as possible.

### *Experiment I*

Twenty-four RIIIf (previously referred to as RIIIb) mice and their litter mate brothers were fed between 6 and 120 hours after birth with vesicular fluid, and four with testis, of $C_3H$ mice. These $C_3H$ males (about 12 months old) were brothers or sons of mammary-tumour-bearing mice.

After being fed, the offspring were returned to their parents until weaning time, after which each litter was left to force-breed between brothers and sisters until up to 12 litters were produced. Males and females were left continuously in the cage until death, and their litters were killed shortly after birth, except when they were killed and eaten by their own parents.

*Experiment II*

(*a*) Six C57 female mice about 48 hours old were fed with fresh testes from $C_3H$ males. (*b*) Six C57 female mice of the same age were fed with fresh salivary gland from the same males used in (*a*). Both groups were force-bred as in Experiment I above.

After 19 months all the mice are alive and none shows any tumour, nor do their C57 mothers, now 26 months old.

TABLE I.—*Fate of RIIIb Female Mice Fed with Vesicular Fluid and Testis from $C_3H$ Males*

| Mouse No. | Age when fed (hours). | Age at death (days). | Total number of litters. |
|---|---|---|---|
| 1 | 6 | 606 | 9 |
| 5 | 12 | 630 | 8 |
| 6 | 12 | 630 | 5 |
| 18 | 12 | 693 | 3 |
| 19 | 12 | 670 | 6 |
| 20 | 12 | 693 | 6 |
| 11 | 24 | 371 | 8 |
| 21 | 24 | 717 | 6 |
| 22 | 24 | 796 | 7 |
| 25 | 24 | 714 | 7 |
| 2 | 48 | 625 | 3 |
| 3 | 48 | 515 | 2 |
| 4 | 48 | 138 | 2 |
| *14 | 48 | 520 | 12 |
| *15 | 48 | 418 (MT) | 9 |
| *16 | 48 | 641 | 5 |
| *17 | 48 | 622 | 4 |
| 26 | 72 | 552 (MT) | 6 |
| 27 | 72 | 823 | 6 |
| 28 | 72 | 826 | 8 |
| 24 | 72 | 564 | 6 |
| 7 | 96 | 784 | 4 |
| 8 | 96 | 564 | 6 |
| 9 | 96 | 776 | 3 |
| 10 | 96 | 336 | 4 |
| 12 | 96 | 663 | 10 |
| 13 | 96 | 461 | 9 |
| 23 | 120 | 687 | 10 |

* Testis fed.
MT = Mammary tumour.

### RESULTS

In the first experiment 2 out of 28 female mice developed a mammary tumour. One (fed with testis) was killed at 418 days bearing an epidermoid carcinoma on the right side at the level of the 4th breast, while the other (fed with vesicular fluid) was killed at 552 days with a fibroadenoma of the right side at the level of the 3rd breast.

### DISCUSSION

Previous experiments with susceptible females borne by supposedly virus-free mothers from virus-carrying fathers suggested that the presence of the father at the time of birth and subsequently might in some way provide a source of infection leading to the development of a mammary tumour in the offspring (Peacock, 1953). But it was shown by Pullinger (1953) that some CBA females reputed to be virus-free were sporadic carriers of the agent. In the present experiment neither parent carried the virus and the only possible source of virus was the vesicular fluid or testes of the high mammary cancer strain used for feeding.

The tumour incidence, 2 out of 28, does not exceed the expected amount seen in RIIIf mice which have lived to a similar age and borne over 5 litters (Pullinger, 1955). Moreover the histology of the tumour, an epidermoid carcinoma and a fibroadenoma with sarcomatous changes in the stroma, differs from the adeno-carcinomatous type characteristic of virus-induced mammary cancer in mice.

Vesicular gland sections stained by the phloxine tartrazine method failed to reveal inclusion bodies, but extensive examination of mammary glands and tumours from high cancer strain did not show either any characteristic structure of this kind (Pullinger, 1956, personal communication), though the mammary gland in the high cancer strain mice is an accepted source of mammary tumour agent.

There is a physiological similarity in the method by which mammary gland and vesicular glands discharge their products.

Biancifiorni, Lotti and Squartini (1954) have described an apocrine secretion of the cellular layer in the male reproductive glands which would allow the discharge of the agent and of normal macromolecules with the shed cells into the lumen of the gland. However the vesicular fluid or testes differ from semen and selection of only one of the components, as in these experiments, might fail to provide an active virus.

In all such experiments the problem of what constitutes an infective dose has to be considered. It has been shown that about 0·1 ml. of milk from a high mammary cancer strain is sufficient to infect susceptible female mice up to 48 hours of age, of high cancer strain ($C_3H$) fostered on C57 (Andervont, Shimkin and Bryan, 1942), or low cancer strain C57 fed with RIII milk (Haagensen and Randall, 1945).

Graff *et al.* (1949) report carcinoma of the breast in mice injected intraperitoneally with particulate fractions obtained by differential centrifugation of high cancer strain milk containing as little as 8 m$\gamma$ of nitrogen. But such quantitative data refer to material of undetermined viral content and Huseby, Barnum and Bittner (1950) noted great variations of infectivity in lactating mammary glands of different mice of the same inbred strain.

In the natural transmission of the mammary tumour virus from mother to offspring the milk provides not only a source of virus but also of oestrogens and possibly other hormones which favour infection. Presumably such hormonal factors are still provided by the milk of the virus-free females of high cancer strain. It was thought therefore that offspring of such RIIIf mothers would provide the ideal subjects for the reception of virus from the $C_3H$ male source.

The negative result of this experiment could be explained by the absence of a virus or its presence in subinfective amount.

### SUMMARY

Oral feeding of vesicular fluid or testes of $C_3H$ mice to RIIIf females did not cause an increase of mammary tumour incidence in excess of what is expected from an average breeding female of this strain.

The author has carried out these investigations while working under a full-time grant from the British Empire Cancer Campaign.

### REFERENCES

ANDERVONT, H. B. AND DUNN, T. B.—(1948) *J. nat. Cancer Inst.*, **8**, 227.
*Idem*, SHIMKIN, M. B. AND BRYAN, W. R.—(1942) *Ibid.*, **3**, 309.
BIANCIFIORNI, C., LOTTI, G. AND SQUARTINI, F.—(1954) *Lav. Anat. Pat., Perugia, XIV*, Fasc. III, p. 249.
GRAFF, S., MOORE, D. H., STANDLEY, W. M., RANDALL, H. T. AND HAAGENSEN, C. D.—(1949), *Cancer*, **2**, 755.
HAAGENSEN, C. D. AND RANDALL, H. T.—(1945) *Cancer Res.*, **5**, 352.
HUSEBY, R. A., BARNUM, C. P. AND BITTNER, J. J.—(1950) *Ibid.*, **10**, 516.
MÜHLBOCK, O.—(1950) *J. nat. Cancer Inst.*, **10**, 861.—(1952*a*) *Ibid.*, **12**, 819.—(1952*b*) *Ibid.*, **13**, 506.
PEACOCK, A.—(1953) *Brit. J. Cancer*, **7**, 352.
PULLINGER, B. D.—(1953) *Ibid.*, **6**, 490.—(1955) *Ibid.*, **9**, 613.